# Health Risk Assessment of Heavy Metal Accumulation in Broiler Chickens and Heavy Metal Removal in Drinking Water using *Moringa Oleifera* Seeds in Lomé, Togo

**DOI:** 10.5696/2156-9614-11.31.210911

**Published:** 2021-08-17

**Authors:** Idrissa Soumaoro, Wéré Pitala, Kissao Gnandi, Tona Kokou

**Affiliations:** 1 Regional Centre of Excellency on Avian Science, University of Lomé, Togo; 2 School of Agronomy, University of Lomé, Togo; 3 Faculty of Sciences, University of Lomé, Togo

**Keywords:** heavy metals, bioaccumulation, chicken, *Moringa oleifera* seeds, consumer health

## Abstract

**Background.:**

Heavy metals are persistent in the environment and can cause bioaccumulation in the food chain. Drinking water contamination by heavy metals can pose a risk to poultry and to human health. The need for affordable, reliable and effective methods of water treatment has led to the use of plants materials, including coagulants such as *Moringa oleifera* seeds, to ensure poultry products are safe for consumers.

**Objectives.:**

The aim of the present study was to investigate the effects of drinking water treatment by *Moringa oleifera* seed on the concentration and distribution of metals such as arsenic (As), cadmium (Cd), chromium (Cr), copper (Cu), nickel (Ni), lead (Pb) in different parts of broilers chickens and theirs effects on consumer health.

**Methods.:**

A total number of 264 one-day old chickens (Cobb-500) were assigned to three treatments, with 22 birds in each treatment and replicated four times: untreated well water (UW), well water treated with *Moringa oleifera* seeds and filtered (MOF) and well water treated with *Moringa oleifera* seeds but unfiltered (MOU). Thirty birds were randomly chosen from different treatments and slaughtered at day 45 of the experiment and samples of livers, kidneys, gizzards and breasts were obtained and analyzed for toxic metals concentrations using atomic absorption spectrophotometry.

**Results.:**

The results indicated that the bioaccumulation of heavy metals was lower in MOF than those in UW and MOU. The target hazard quotient (THQ) for individual metals was below acceptable limits except for As and Pb in UW and MOU. The carcinogenic risk (TR) was estimated for each metal due to consumption of different types of chicken.

**Conclusions.:**

*Moringa oleifera* seeds are an environmentally friendly natural coagulant and able to treat water containing undesirable heavy metal concentrations and ensure that poultry meat is safe for consumers.

**Competing Interests.:**

The authors declare no competing financial interests.

**Ethics Approval.:**

This study was approved by the ethics Committee of the University of Lomé-Togo.

## Introduction

The poultry industry is one of the largest and fastest growing agro-based industries in the world.^1^

Chicken meat has high biological value, as it contains essential amino acids required to promote human growth and health. Despite its nutritional benefits, the quality of poultry meat may be affected by the contamination of toxic metals through various anthropogenic activities.^2^ Toxic metal contamination of meat poses a risk to human health, because as metal accumulation increases in the food chain this can cause various adverse health effects. The main sources of metals in chicken include contamination of poultry feeds, drinking water and processing.^3^ The toxic effects of heavy metals to the chickens include feed refusal, loss of weight, low digestibility, organ failure and death, and for humans they include kidney and liver damage, anemia, alteration of central nervous system and cancer.^4^ Many heavy metals are used as trace elements and feed additives in poultry feed. These metals are common in the environment. Some of these (iron (Fe), copper (Cu), manganese (Mn), zinc (Zn)) are essential for good health, however, others (arsenic (As), mercury (Hg), lead (Pb), cadmium (Cd)) are harmful to health.^5^ Arsenic, Cd, chromium (Cr), nickel (Ni) and Pb pose the greatest risk and have deleterious impacts on human health. Heavy metals accumulation in chicken meat is a result of increased industrialization without pollution prevention measures, particularly in drinking water.^6^ Few studies have investigated toxic metal accumulation and contaminated diets in poultry production to assess the potential risk to human health.^7^–^8^ These health risks include neurological deficits, developmental deficits in children, high blood pressure, as well as severely disrupted enzyme activity.^9^ Cadmium is toxic to virtually every system, accumulates within the kidney and liver and can cause kidney dysfunction, skeletal damage, prostate cancer, and mutation.^10^–^11^ Chromium can exist in different oxidation states, is an essential micro element and plays an important role in nutrition. Chromium (III) is a cofactor of insulin, which is involved in glucose, lipid and protein metabolism. However, the hexavalent form of Cr is carcinogenic.^12^ The need for simple, reliable and effective methods of water treatment has led to the use of plant materials, including the seeds of *Moringa oleifera* as coagulants. *Moringa oleifera* is a natural coagulant that can replace aluminium sulphate (alum), which is used globally.^13^ One study found that *Moringa oleifera* seed removed 95% of Cu, 93% of Pb and 70% of Cr. 5 Another study reported that *Moringa oleifera* seed removed 85% of Cd, 81% of Cr, 76% of Ni in India.^14^

The use of *Moringa oleifera* seeds to remove heavy metals is a sustainable, low cost, locally available, simple, reliable, acceptable, and eco-friendly method suitable for developing countries where a large proportion of the population uses contaminated water for drinking purposes.^9^ Thus, the objective of the current study was to evaluate heavy metals removal in drinking water using *Moringa oleifera* seeds on heavy metal accumulation in broiler chickens.

**Abbreviations**
*MOF*: Well water treated with *Moringa oleifera* seeds and filtered
*MOU*: Well water treated with *Moringa oleifera* seeds and unfiltered
*THQ*: Target hazard quotient
*TR*: Target cancer risk
*USEPA*: United States Environmental Protection Agency
*UW*: Untreated well water

## Methods

*Moringa oleifera* seeds were harvested when they were fully matured from a rural area of Lomé (Adidogomé), Togo. This was determined by observing if there were any cracked pods on the plants. The plucked pods were cracked to obtain the seeds which were air-dried at 40°C for two days. The seeds were blended to obtain a fine powder.^15^ The fine powder was then analyzed for heavy metals contamination. All materials and tools used were sterilized.

### Water treatment

The water treatment was performed by taking 2 g of seed powder and mixing it with 1 L of well water.^16^ For well water treated with *Moringa oleifera* seeds and filtered (MOF), the aqueous solution was filtered through a muslin cloth and strainer for purification. All water treatments were done at room temperature and allowed to stand for two hours. The samples of water for each treatment were collected for analysis for physicochemical and heavy metals.

### Birds and experimental design

Due to the absence of an animal care committee available at the University of Lomé at the time of this research, the present study was conducted under the supervision of the research team leader following the guidelines of the Canadian Council on Animal Care (2009),^17^ and approved by the Ethics committee of the University of Lomé-Togo.

This experiment was conducted at the poultry unit of the Regional Centre of Excellence in Poultry Science, University of Lomé, Togo. The house was an open-sided poultry house. A total of 264, one-day old, unsexed commercial broiler chicks (Cobb-500) were used. The chicks were randomly assigned to three (3) experimental treatments allocated to 4 replications of 22 birds each in a floor spacing of 1.20 m × 1.75 m with wood shavings as litter material. The treatments included untreated well water (UW), MOF, and treated well water with *Moringa oleifera* seeds but unfiltered (MOU). The aqueous solution was filtered through a muslin cloth and strainer to be purified. The lighting program consisted of full-time light for the first 3 days, followed by 20 h of light until day 7 and 16 h of light thereafter. The same diet was fed to all birds which were formulated to meet or exceed the requirements of broiler chickens.^6^ Routine and occasional management and vaccinations for Newcastle disease and coccidiosis were carried out. Water and feed were provided *ad libitum* during the experimental period.

At six weeks old, ten chickens were picked at random from each treatment and slaughtered. Livers, kidneys, breasts and gizzards were taken to determine heavy metal residues.

### Sample digestion

Samples (breast, kidney, liver and gizzard) were collected and stored at −18°C and digested as soon as possible. Chicken samples were cut into small, 2 g (wet weight) pieces. Each sample was drawn into a quick-fit conical flask and a mixture of 15 ml concentrated nitric acid and perchloric acid (4:1 vol/vol) was added. The mixture was then digested in a condenser and stirred at 80°C for 2 hours until the solution became colorless.^7^,^18^,^19^ Afterwards, the sample was removed and allowed to cool. The mixture was filtered with Whatman grade 42 filter papers from PromaLab. The conical flask was then rinsed with double distilled water and the filtrate was transferred into a volumetric flask and finally diluted with 25 ml of distilled water. Trace amounts of residue remained on the filter paper.

### Sample analysis

Samples were analyzed using an atomic absorption spectrometer (Thermo Electron S series). The data were analyzed with the Graph Pad Prism 5 statistical software package. One-way analysis of variance (ANOVA) was used to analyse heavy metal concentrations. Pearson bivariate correlation was used to evaluate inter-element associations.

### Health risk estimation

Target hazard quotient (THQ) is an estimate of the risk level (non-carcinogenic) due to pollutant exposure. To estimate the human health risk from contaminated chicken, the THQ was calculated using the United States Environmental Protection Agency (USEPA) Region III Risk-Based Concentration Table 8.^20^ Equation 1 was used for estimating THQ:

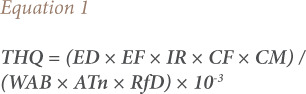



Where THQ is the target hazard quotient, EF is the exposure frequency (365 days/year), ED is the exposure duration (30 years for non-cancer risk as suggested by the USEPA), IR is the ingestion rate of chicken tissue (300 g/person/day), CM is the metal concentration in chicken (mg/kg, wet weight), WAB is the average body weight (70 Kg), ATn is the average exposure time for non-carcinogens (EF×ED) (365 days/year) for 30 years (ATn=10,950 days) as used for characterizing non-cancer risk, and RfD is the reference dose of the metal (an estimate of the daily exposure to which the human population may be continuously exposed over a lifetime without an appreciable risk of deleterious effects).^21^–^22^

### Hazard index

The hazard index from THQs is expressed as the sum of the hazard quotients, Equation 2.^21^


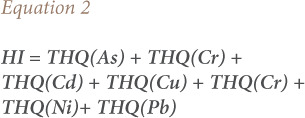


### Target cancer risk

Target cancer risk (TR) was used to indicate carcinogenic risk. The method to estimate TR is also provided in the USEPA Region III Risk-Based Concentration Table 6.^20^ Equation 3 was used for estimating TR.


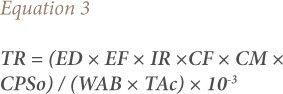


Where TR is the target cancer risk, CM is the metal concentration in chicken (mg/kg, wet weight), IR is the chicken ingestion rate (g/day), CPSo is the carcinogenic potency slope, oral (mg/kg body weight/day), and ATc is the average time for carcinogens (365 days/year for 70 years).^21^ The CPSo values (mg/kg body weight/day) are 1.5, 0.38, 0.5, 0.7, 1.7 and 0.0085 for As, Cd, Cr, Cu, Ni and Pb respectively, thus TR values were calculated for intake of these metals.

## Results

The concentration of heavy metals detected in each part of the chickens in the filtered group (MOF) are indicated in Tables 2, 3 and 4. The results showed the concentrations of metals in the filtered group (MOF) in the following order: As > Cr > Cu > Ni > Cd > Pb.

**Table 1:** Age and Weight of Different Treatments of Broiler Chickens

**Type of water given to poultry**	**Age (weeks)**	**Weight**
Untreated well water (UW)	6 weeks	1.7–2.04 kg
Treated with *Moringa oleifera* seeds and filtered (MOF)	6 weeks	1.9–2.05 kg
Treated with *Moringa oleifera* seeds but unfiltered (MOU)	6 weeks	1.3–1.8 kg

**Table 2:** Concentrations of Arsenic and Cadmium Across Treatment Samples Compared with World Health Organization Standards

**Treatment**		**UW**				**MOU**				**MOF**			

Chicken part		B	L	K	G	B	L	K	G	B	L	K	G
**As (mg/k)**	Max	103	166	794	50.1	18.4	31.4	713	42.9	43.8	435	196	424
	Min	99.5	28.4	281	11.7	9.82	22.9	451	15.1	12.9	320	131	376
	Mean	101*	66.8*	502*	33.3*	12.2*	28*	564*	32.7*	22.7*	380*	161*	391*
	Med	99.7	58.5	462	40.9	11.3	28.6	561	34.8	20.1	403	158	384
	SD	1.11	15.0	97	2.01	1.00	1.0	40.3	1.01	3.35	16.5	7.69	6.16

**Cd (mg/K)**	Max	0.21	0.09	0.12	0.050	0.02	0.07	0.33	0.04	0.04	0.67	0.44	.04
	Min	0.02	0.03	0.01	0.010	0.01	0.02	0.13	0.02	0.01	0.03	0.18	0.02
	Mean	0.04*	0.06	0.06*	0.03*	0.01*	0.036	0.17*	0.03*	0.02*	0.158	0.266*	0.03*
	Med	0.02	0.07	0.065	0.040	0.02	0.035	0.13	0.030	0.02	0.07	0.23	0.03
	SD	0.0234	0.012	0.055	0.0120	0.0014	0.006	0.0395	0.003	0.003	0.075	0.0358	0.0021
**WHO/FAO Standard^21^**		AS (mg/Kg) 0.1				Cd (mg/Kg) 0.3							

^*^indicate statistically significant differences (p<0.05).

Abbreviations: B, breast; L, liver; K, kidney; G, gizzard; MOF, well water treated with *Moringa oleifera* seeds and filtered; MOU, well water treated with *Moringa oleifera* seeds but not filtered; UW, untreated water

**Table 3:** Concentrations of Chromium and Copper Across Treatment Samples Compared with World Health Organization Standards

**Treatment**		**UW**				**MOU**				**MOF**			

Chicken part		B	L	K	G	B	L	K	G	B	L	K	G
**Cr (mg/Kg)**	Max	0.91	0.71	1.26	0.98	1.21	4.34	3.87	1.47	1.94	12.8	14.2	2.52
	Min	0.73	0.43	0.44	0.4	0.65	2.13	2.89	1.34	1.46	11.6	13.2	2.26
	Mean	0.80*	0.58*	0.86*	0.73*	0.99*	3.0*	3.15*	1.43*	1.75*	12.3*	13.8*	2.39*
	Med	0.815	0.56	0.865	0.77	1.07	2.99	3.06	1.44	1.76	12.4	13.9	2.39

**Cu (mg/Kg)**	SD	0.0205	0.033	0.118	0.0762	0.0732	0.251	0.113	0.0139	0.05	0.13	0.119	0.0353
	Max	0.81	9.49	14.1	8.03	11.6	11.3	4.66	6.38	1.60	9.93	15.1	7.27
	Min	0.68	4.68	11.1	5.54	9.88	2.22	3.84	5.02	0.54	8.17	9.98	6.97
	Mean	0.76*	7.19*	12.6*	7.80*	10.5*	3.90*	4.19*	6.01*	1.12*	9.06*	12.2*	7.24*
	Med	0.78	7.45	12.5	8.03	10.4	2.97	4.06	6.38	1.1	9.15	12.1	7.27
	SD	0.0157	0.668	0.519	0.577	0.242	0.193	1.07	0.123	0.228	0.135	0.893	0.0663
WHO/FAO Standard^21^		**Cr (mg/Kg) 1.0**								**Cu (mg/Kg) 0.4**			

^*^indicate statistically significant differences (p<0.05).

Abbreviations: B, breast; L, liver; K, kidney; G, gizzard; MOF, well water treated with *Moringa oleifera* seeds and filtered; MOT, well water treated with *Moringa oleifera* seeds but not filtered; UW, untreated water

**Table 4:** Concentrations of Nickel and Lead Across Treatment Samples Compared with World Health Organization Standards

**Treatment**		**UW**				**MOU**				**MOF**			

Chicken part		B	L	K	G	B	L	K	G	B	L	K	G
**Ni (mg/Kg)**	Max	0.42	0.36	4.3	0.7	0.84	0.95	7.78	0.36	0.71	9.08	7.45	1.35
	Min	0.13	0.06	0.78	0.26	0.05	0.33	2.81	0.12	0.05	0.18	2.21	0.2
	Mean	0.276*	0.21*	1.79*	0.441*	0.399*	0.51*	4.09*	0.225*	0.266*	2.32*	4.95*	0.561*
	Med	0.27	0.22	1.2	0.425	0.42	0.465	3.61	0.185	0.22	1.07	4.81	0.485
	SD	0.0395	0.034	0.427	0.0532	0.0736	0.069	0.562	0.0363	0.081	1.09	0.62	0.122

**Pb (mg/Kg)**	Max	0.72	0.42	1.35	0.35	0.34	0.67	1.87	0.42	0.22	0.43	1.73	0.28
	Min	0.06	0.06	0.18	0.07	0.18	0.27	0.13	0.18	0.11	0.15	0.77	0.05
	Mean	0.23*	0.23*	0.9*	0.17*	0.254*	0.438*	1.28*	0.28*	0.184*	0.25*	1.22*	0.14*
	Med	0.14	0.24	1.04	0.16	0.245	0.415	1.44	0.27	0.19	0.21	1.14	0.125
	SD	0.084	0.054	0.256	0.043	0.02	0.049	0.244	0.03	0.012	0.061	0.15	0.032
WHO/FAO Standard^21^		**Ni (mg/Kg) 0.5**				**Pb (mg/Kg) 0.5**							

^*^indicate statistically significant differences (p<0.05).

Abbreviations: B, breast; L, liver; K, kidney; G, gizzard; MOF, well water treated with *Moringa oleifera* seeds and filtered; MOU, well water treated with *Moringa oleifera* seeds but not filtered; UW, untreated water

The concentration of heavy metals detected in each part of the birds in the untreated group (UW) are indicated in Tables 2, 3 and 4. The results showed that the concentrations of metals from four parts of untreated groups occurred in the following order: As > Cu > Cr > Cd > Pb > Ni.

The concentration of heavy metals detected in each part of the birds in the unfiltered group (MOU) are indicated in Table 2, 3 and 4. The results showed the concentrations of metals from four parts of the unfiltered group in the following order: As > Cu > Ni > Cr > Pb > Cd.

The results showed that the highest concentrations of As and Pb were found in the unfiltered group (MOU), whereas the highest Cu was observed in the untreated group (UW), and the highest Cd, Cr and Ni were found in the filtered group (MOF).

The highest concentration of As, Cu and Cd were found in the kidney sample and the lowest in the breast. The highest concentration of Pb was found in the kidney sample and the lowest in the gizzard. The highest concentration of Ni was found in the kidney sample and the lowest in the liver.

**Figure 1:**
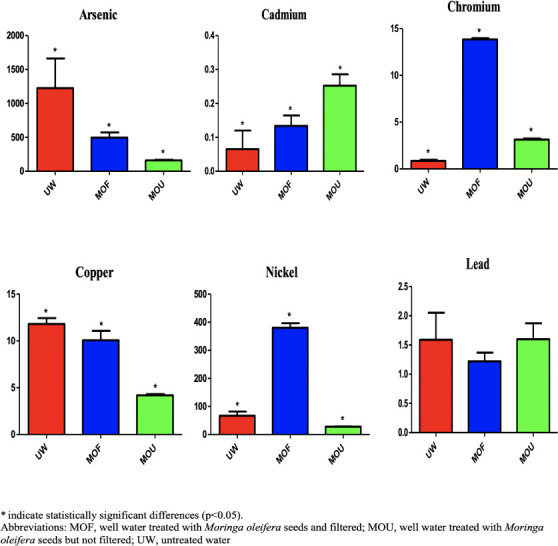
Average metals concentrations in breast meat samples across treatment types

The average metal concentrations in the breast of different types of treatments are shown in Figure 1. Arsenic concentrations ranged from 9.82 to 103 mg/kg. The highest concentrations were found in the untreated group (UW). The arsenic content in the samples decreased in the order of untreated > filtered > unfiltered. According to the World Health Organization (WHO) standard, the maximum permissible limit of As is 0.1 mg/kg.^21^ All of the concentrations in the studied samples were higher than the maximum permissible limit.

**Figure 2:**
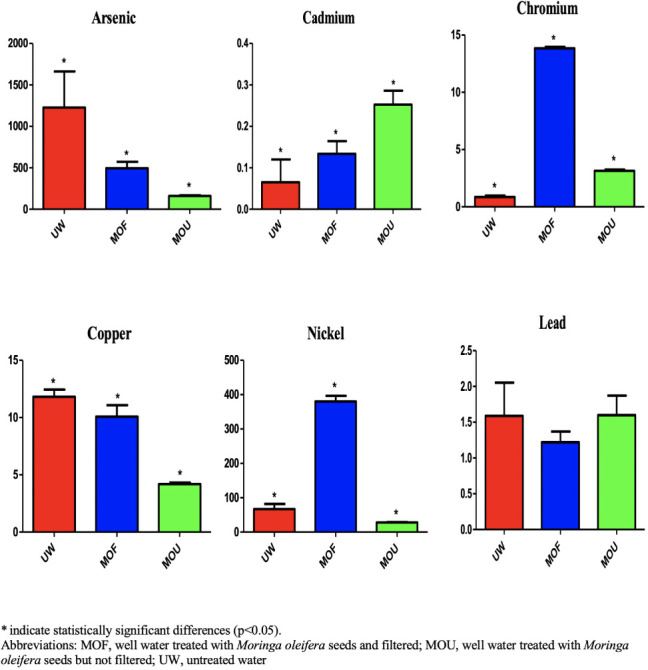
Average metal concentrations in kidney samples across treatment types

**Figure 3:**
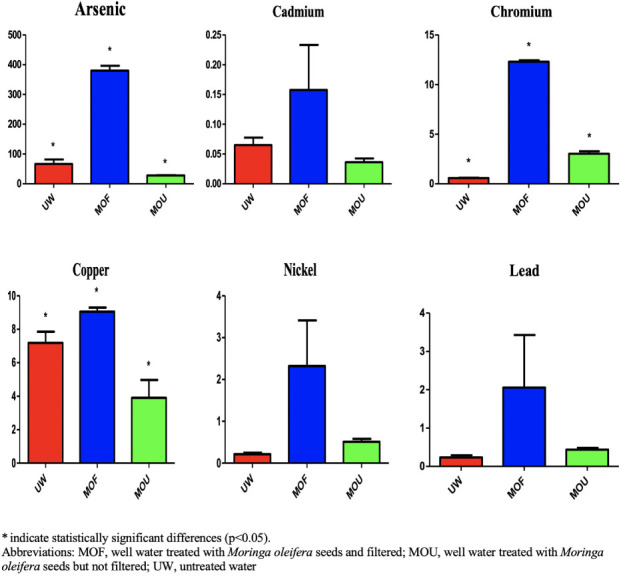
Average metals concentrations in liver samples across treatment types

**Figure 4:**
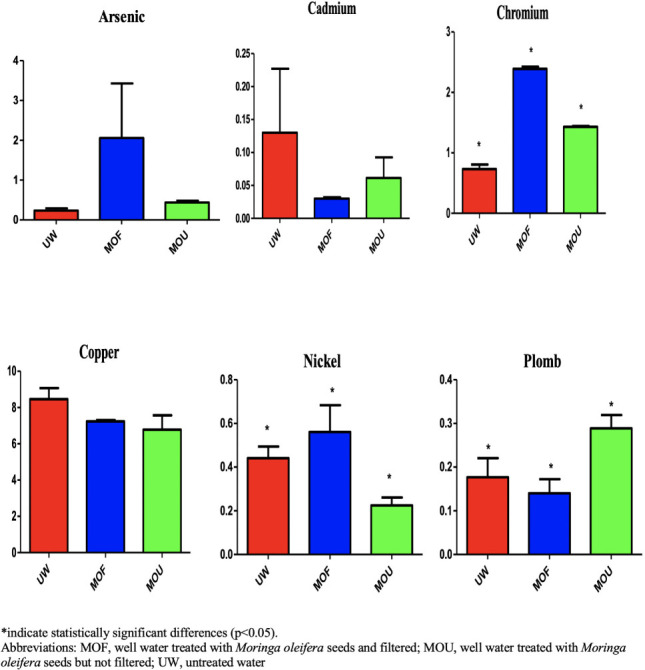
Average metals concentrations in gizzard samples across treatment types

Cadmium concentrations ranged from 0.01–0.46 mg/kg. The highest concentration was found in the untreated group (UW). The Cd content in the samples decreased in the order of untreated > filtered > unfiltered. According to the WHO standard, the maximum permissible limit of Cd is 0.3 mg/kg.^21^ In the studied sample, the concentration of cadmium in the untreated group was higher than the maximum permissible limit, although Cd concentrations were within the permissible limit both in the filtered and unfiltered groups.

Chromium (III) is an essential element at low concentrations, although the hexavalent form of Cr is carcinogenic.^23^ The recommended daily intake for adults is between 0.02 and 0.5 mg/day.^21^ In the present study, the concentration of Cr ranged from 0.65–1.94 mg/kg. The highest concentration was found in the filtered group, followed by the unfiltered group and untreated group. According to the WHO, the maximum permissible limit of Cr in chicken meat is 1 mg/kg. The concentration of Cr in the filtered group is much higher than the maximum permissible limit, although concentrations in both the untreated and unfiltered groups were within permissible limits.

The Cu concentrations found in the analyzed samples ranged from 0.54–11.6 mg/kg. The highest concentration of Cu was found in the unfiltered group. The order of Cu concentration was unfiltered > filtered > untreated. According to the WHO, the maximum permissible limit of Cu is 0.4 mg/kg.^21^ The concentration in the unfiltered group was much higher than the maximum permissible limit.

The tolerance levels of Ni in children and adults are 7 mg/days and 40 mg/days, respectively.^4^ The highest concentration of Ni was found in the unfiltered group (0.84 mg/kg), and the lowest was in the filtered group (0.05). According to the WHO, the maximum permissible limit of Ni is 0.5 mg/kg.^21^ In this studied sample, the concentration of Ni in the unfiltered sample was higher than the maximum permissible limit.

The Pb concentrations found in the analyzed samples ranged from 0.06 to 0.18 mg/kg. The highest concentration of Pb was found in the unfiltered group. The order of Pb concentration was unfiltered > filtered > untreated. According to the WHO, the maximum permissible limit of Pb is 0.5 mg/kg.^21^ The concentrations of Pb in the present study were much higher than maximum permissible limits.

### Correlation coefficient of metals

The correlation coefficient r measures the strength and direction of a linear relationship between two variables on a scatter plot. The value of r is always between +1 and −1. A correlation value of +1 indicates a perfect positive linear relationship. As one variable increases in value, the other variable also increases. A correlation value of −1 indicates a perfect negative linear relationship. As one variable increases in value, the other variable decreases in value via an exact linear rule. A correlation greater than 0.8 is generally described as strong, whereas a correlation less than 0.5 is generally described as weak. The Pearson coefficient was used to calculate the correlation between trace metals concentrations and to establish the mutual influence in the bioaccumulation process.

Table 5 shows a strong positive correlation between Ni-Cd (r^2^=0.99), Pb-Cd (r^2^=0.98), Pb-Ni (r^2^=0.97), Cu-As (r^2^=0.82) and a positive correlation between Cr-As (r^2^=0.58) in the untreated group. Table 6 shows a significantly high negative correlation for all metals and a positive correlation between Ni-Cr (r^2^=0.60) in the filtered group. Table 7 shows a weak positive correlation between Cr-As (r^2^=0.45), Cu-Cd (r^2^=0.47), Ni-AS (r^2^=0.37), Pb-Cu (r^2^=0.44) and a significantly high negative correlation between Cu-AS, Ni-Cd, Ni-Cr, Ni-Cu, Pb-Cr in the unfiltered group.

**Table 5:** Correlation Matrix Between Metal Concentrations in theUntreated Group (UW)

**Metals**	**As**	**Cd**	**Cr**	**Cu**	**Ni**	**Pb**
**As**	1					
**Cd**	0.116	1				
**Cr**	0.580*	0.032	1			
**Cu**	0.827**	0.223	0.2354	1		
**Ni**	0.182	0.994**	0.1164	0.2672	1	
**Pb**	−0.030	0.988**	−0.0502	0.1085	0.974**	1

^*^Correlation is significant at 0.05 level (two-tailed)

^**^ Correlation is significant at 0.01 level (two-tailed)

**Table 6:** Correlation Matrix Between Metal Concentrations in the Filtered Group (MOF)

	**As**	**Cd**	**Cr**	**Cu**	**Ni**	**Pb**
**As**	1					
**Cd**	0.170	1				
**Cr**	0.123	0.204	1			
**Cu**	0.484	−0.329	−0.1833	1		
**Ni**	−0.350	0.609*	0.3980	−0.5910	1	
**Pb**	−0.035	−0.8627	−0.0585	0.2809	−0.7138	1

Correlation is significant at 0.05 level (two-tailed)

^**^ Correlation is significant at 0.01 level (two-tailed)

**Table 7:** Correlation Matrix Between Metal Concentrations in the Unfiltered Group (MOU)

	**As**	**Cd**	**Cr**	**Cu**	**Ni**	**Pb**
**As**	1					
**Cd**	−0.071	1				
**Cr**	0.459	0.052	1			
**Cu**	−0.693	0.472	−0.056	1		
**Ni**	0.379	−0.167	−0.183	−0.742	1	
**Pb**	−0.701	0.459	−0.746	0.441	0.048	1

^*^Correlation is significant at 0.05 level (two-tailed)

^**^ Correlation is significant at 0.01 level (two-tailed)

**Table 8:** Target Hazard Quotient of Trace Metals from Consumption Across Treatment Types

**Metals**	**Rfd (mg/kg/day)**	**Target hazard quotient^20^**		

		MOF	UW	MOU
As	0.0003	0.236	1.053	0.127
Cd	0.001	0.381	0.724	0.278
Cr	0.003	0.054	0.025	0.031
Cu	0.03	0.234	0.017	0.025
Ni	0.02	0.0024	0.002	0.003
Pb	0.003	0.959	1.199	1.324

Abbreviations: MOF, well water treated with *Moringa oleifera* seeds and filtered; MOU, well water treated with *Moringa oleifera* seeds but not filtered; UW, untreated water; Rfd, reference dose of metal, HI, hazard index.

**Table 9:** Target Cancer Risk of Trace Metals from Consumption Across Treatment Types

**Metals**	**CPSo (mg/kg)**	**Target cancer risk^21^**		

		MOF	UW	MOU
As	1.5	0.236	1.0143	0.1225
Cd	0.38	6 10^−5^	10^−3^	4 10^−4^
Cr	0.5	5 10^−3^	2 10^−3^	3 10^−3^
Cu	0.7	5 10^−3^	3 10^−3^	4 10^−2^
Ni	1.7	3 10^−3^	3 10^−3^	4 10^−3^
Pb	0.0085	1.04 10^−5^	1.3 10^−5^	1.44 10^−5^

Abbreviation: MOF, well water treated with *Moringa oleifera* seeds and filtered; MOU, well water treated with *Moringa oleifera* seeds but not filtered; UW, untreated water; CPSO, Carcinogenic potency slope, oral exposure.

The THQ values of the analyzed chicken samples were less than 1 for all individual trace metals except for As, Pb in the untreated group (UW) and Pb in the unfiltered group (MOU). The results indicate that ingestion of As and Pb through consumption of untreated chicken should have high non-carcinogenic toxic effects.

The results of the current study revealed that TR values of the analyzed samples for some traces metals were intolerable (As, Ni, Cu and Cr) in three groups, intolerable for Cd in the untreated (UW) group, but considered satisfactory for the filtered (MOF) and unfiltered (MOU) group, indicating a possible cancer health risk from consumption. Trace metals for Pb were within acceptable limits, indicating no cancer health risk for Pb.

## Discussion

The concentration of As levels recorded in breast samples (22.7, 12.2 and 101 mg/kg, respectively, for MOF, MOU and UW) were higher than those reported by Akan *et al.* (0.3 mg/kg).^2^ The removal of heavy metal concentrations can be explained by the process of coagulation with coagulants present in *Moringa oleifera*. The mechanism of interaction between the *Moringa oleifera* proteins and heavy metals was ion adsorption and charge neutralization. *Moringa oleifera* seed powder has been termed a potential heavy metal removing agent due to its oxygen and nitrogen donating carboxylate and amino groups.^16^ *Moringa oleifera* is limited to the adsorption surfaces. This is because *Moringa oleifera* is a cationic polyelectrolyte of short chain and low molecular weight. Adsorption describes attachment of ions and molecules from seed protein by means of a specific mechanism. Metal adsorption occurs due to the high protein content of the seeds. The flocculation activities of *Moringa oleifera* seeds are based on the electrostatic patch charge mechanism.^16^ The process of up flow roughing filtration separates the flocs formed by *Moringa oleifera* seed coagulant. This is similar to the results of Ravikumar and Sheeja, who reported that *Moringa oleifera* seed removed 95% of Cu, 93% of Pb and 70% of Cr. Sharma *et al.* (2007) also reported that *M. oleifera* seed removed 85% of Cd, 81% of Cr, 76% of Ni in India.^14^–^16^

Okeye reported As concentrations in internal organs of broiler chicken in the range of (0.053–0.119 mg/g).^19^ Several studies have shown that inorganic arsenic can cause lung, bladder, liver, kidney, prostate and skin cancer. Emerging science also shows that inorganic arsenic may harm pregnant women and fetuses. Arsenic has been shown to cross the placenta to the foetus and has been found in breast milk. Chronic exposure to arsenic has been shown to affect child development by lowering IQ scores.^12^

The concentration of Cd recorded in the untreated group (UW) was higher than the Cd contents (2.67–4.33 mg/Kg) found in chicken in Bangladesh in a study by Rahman *et al.*^6^ The Cd level in the filtered and unfiltered groups were similar to levels reported in previous studies 0.012–0.227 mg/kg and 0.45–2.23 mg/kg in different tissues of chicken, respectively. 6–7

The Cr concentrations recorded in this study in the filtered group (MOF) were higher than those reported by Iwegbue *et al.* (0.01–3.34 mg/kg).^4^ The mean concentration of Cr (0.58–13.1 mg/kg) was higher than concentrations reported in northern Nigeria (2.55 mg/kg, Orisakwe *et al.*, 2017).^22^ In general, the concentration of total Cr ranged from 0.01 to 1.3 mg/kg. This high concentration of Cr in the filtered group (MOF) could be explaining by feed contamination by Cr.

The concentrations of copper recorded in this study were higher than the results obtained by Alturiqi and Albedair (2.31 to 7.79 mg/kg), those recorded by Mahmoud Elsharawy (0.15–1.16 μg/g), Orisakwe *et al.* (1.012 mg/kg) and Menezes *et al.* (0.65–0.84 mg/kg).^23^,^24^,^25^,^26^

Copper concentrations in meat increase with chicken age and depend on the concentration of Cu in feed.^27^ Although Cu is an essential nutritional element, high intakes can cause health problems such as liver and kidney damage (Agency for Toxic Substances and Diseases Registry, 2004).^28^ Copper is an important constituent in a number of different enzymes.

Nickel plays an important role in human health; however, high levels of Ni content may result in serious respiratory distress and cancer. The mean Ni content in chicken in this study (0.225–4.95 mg/kg) was higher than the values reported in Derata, northern Nigeria (1.012 mg/kg).^29^

The maximum concentration of Ni obtained (UW) in the current study was higher than previous studies reported by Wang *et al.* (76.5 μg/kg), Zahurul *et al.* (0.49 mg/kg) and Yuanan Hu *et al.*^30^,^31^,^32^

The lead concentrations recorded in this study were similar to the value reported by Yeasmin *et al.* (0.228–0.290 mg/kg) 33. The high concentration of lead in the muscle (UW and MOU) indicates long term bioaccumulation. High levels of Pb in poultry meat result mainly from contamination of feed or water sources. Mariam *et al.* (2004) reported mean levels of 3.15 mg/kg for lead in poultry meat.^34^

### Health risk estimation

The risk associated with the carcinogenic effects of a target metal is expressed as the probability of contracting cancer over a lifetime of 70 years. The THQ estimated for the individual metals via consumption of different treatments of chicken is shown in Table 8. If the THQ value is less than 1, the risk of non –carcinogenic toxic effects are assumed to be low.^35^–^36^

Target cancer risk values exceeding 1×10^−4^ are regarded as intolerable a risk value lower than 1×10^−6^ is regarded as safe, and risk values between 1×10^−4^ and 1×10^−6^ are considered satisfactory^37^ and acceptable according to the USEPA.^20^

## Conclusions

The results showed that the concentrations were lower in the filtered (MOF) group than the untreated (UW) and unfiltered groups (MOU), although the concentrations of As, Cd, Cu, Ni and Pb in the analyzed samples were higher than the maximum permissible level (World Health Organization) for the untreated and unfiltered groups. The lower concentration of As, Cd, Cu, Ni and Pb in different parts of chicken in the filtered (MOF) group indicate the importance of heavy metals removal by *Moringa oleifera* seeds. The high concentration in different parts of the untreated (UW) group may be due to metals inadvertently entering the food chain. Arsenic, Cd, Cu, Ni and Pb were higher than permissible maximum levels and indicate a health risk. High contamination of As, Cd and Pb may be caused by drinking water contamination. High concentrations of Cr, Cu and Ni in MO may be a source of contaminated poultry feed. Among the three treatments investigated, the treated and filtered (MOF) groups appeared to be comparatively less contaminated with metal traces than other groups. The target hazard quotient values of the analyzed samples across the three treatments were within permissible limits except for As and Pb in the untreated (UW) and unfiltered (MOU) groups, indicating high non-carcinogenic toxic effects. Target cancer risk values of the analyzed samples for As, Ni, Cu and Cr were intolerable, indicating cancer health risk via consumption of chicken. *Moringa oleifera seeds* are an environmentally friendly natural coagulant suitable for the treatment of water containing undesirable heavy metal concentrations, ensuring that poultry meat is safe for human consumption.
